# Should sagittal osteotomy line be parallel to tibial posterior slope in high tibial osteotomy?

**DOI:** 10.1186/s12891-018-2257-6

**Published:** 2018-09-19

**Authors:** Y. Akamatsu, H. Kobayashi, M. Tsuji, S. Nejima, K. Kumagai, T. Saito

**Affiliations:** Department of Orthopaedic Surgery, Yokohama City University, School of Medicine, 3-9, Fukuura, Kanazawa-ku, Yokohama, 236-0004 Japan

**Keywords:** Open wedge high tibial osteotomy, 3 dimensional computed tomography, Hinge position, Osteotomy plane, Tibial posterior slope

## Abstract

**Background:**

The reason why the osteotomy line in the sagittal view should be parallel to the medial tibial posterior slope in open wedge high tibial osteotomy (OWHTO) remains unclear. In addition, previous study reported that a posterolateral hinge position led to an increase in tibial posterior slope (TPS) after OWHTO. Our aims were to examine the relationships between angles among the tibial plateau and osteotomy planes or the hinge point and the change in TPS, and the location of the hinge position after OWHTO using three-dimensional computed tomography (3DCT). We hypothesized that the sagittal angle between the tibial plateau and osteotomy planes with an anterior-widening proximal tibial fragment resulted in increased TPS, and the hinge position located posterolaterally.

**Methods:**

Preoperative planning anticipated a weight-bearing line ratio of 62% on the radiograph. The anterior gap was 67% of the posterior gap in OWHTO. We identified the tibial plateau and upper and lower osteotomy planes on 3DCT of 82 patients with symptomatic medial osteoarthritic knee after OWHTO. The osteotomy plane angles between the tibial plateau and upper osteotomy planes, and opening gap angles between both osteotomy planes in the coronal and sagittal views were measured. The anteroposterior (AP) and lateral hinge position was displayed as a percentage on the upper osteotomy plane. We assessed the relationships among them.

**Results:**

The TPS significantly increased after OWHTO (*p* = 0.002). There was no significant difference between the sagittal osteotomy plane angle and the change in TPS. The sagittal opening gap angle and the AP hinge position ratio were significantly correlated with the change in the TPS (*r* = 0.477 *p* < 0.001 and *r* = − 0.342, *p* = 0.002, respectively). The hinge position was located a mean of 16.0% from the lateral and 48.6% from the posterior tibial edge in the upper osteotomy plane.

**Conclusions:**

Contrary to our expectation, the osteotomy plane did not need to be parallel to the tibial plateau plane in the sagittal view. However, the osteotomy gap should be rectangular in the sagittal view. The hinge position located nearly in the center of the sagittal view.

## Background

A surgical technique that the osteotomy line in the sagittal view should be parallel to the medial tibial posterior slope (TPS) in open wedge high tibial osteotomy (OWHTO) is recommended [1–4]. Lee et al. reported that the mean sagittal osteotomy plane angle, which is angle between the tibial plateau and upper osteotomy planes in the sagittal view, with an anterior-widening proximal tibial fragment on the radiograph was 15.1°, and 87.1% of knees had an anterior-widening proximal tibial fragment. They showed that the sagittal osteotomy plane angle was positively correlated with the change in TPS [4]. Regarding the control of TPS during surgery, two reports have suggested that the opening gap ratio, which was defined as the ratio of an anteromedial opening gap to a posteromedial opening gap, of 1/2 or 2/3 will minimize the change in TPS [2, 5]. The change in TPS may be controlled by intraoperatively measuring the TPS using two Kirschner wires [6, 7], lateral fluoroscopy projection, and a navigation system [2]. However, despite these techniques, the degree of change in TPS after OWHTO still varies [7].

Two previous studies have tried to investigate the relationship between the hinge position and the change in TPS after OWHTO, but have a small number of cases, which was one study to 12 knees using cadavers [8] and another to 19 knees using three-dimensional computed tomography (3DCT) [9]. They concluded that the hinge position affected the change in TPS, and a posterolateral hinge position led to an increase in TPS after OWHTO.

Our aims were to examine the relationships among the osteotomy plane, opening gap, and hinge position, and to determine the location of hinge position using 3DCT after OWHTO. We hypothesized that the sagittal osteotomy plane angle with an anterior-widening proximal tibial fragment resulted in increased TPS and the hinge position located posterolaterally.

## Methods

### Patients

This was a retrospective analysis of prospective collected data. One surgeon performed all OWHTO between April 2012 and March 2014. We retrospectively analyzed the radiographs and CT images. Inclusion criteria were patients with medial knee OA, an anatomical femorotibial angle (aFTA) of ≦185°, and a flexion contracture of ≦15°. Exclusion criteria were patients with a patellofemoral symptom, an anterior or posterior cruciate ligament insufficiency, and a lateral tibiofemoral joint space narrowing on radiograph. We collected data from 87 patients. Two cases had lateral tibial plateau fracture during OWHTO, and 3 cases did not have all of their radiographs and CT images examined until 2 years postoperatively. Therefore, 5 patients were totally excluded and the final total was 82 included patients. No patients had an extra-articular deformity, or an ipsilateral hip or ankle OA.

### Radiography

The radiographs were projected using a Fuji computed radiography system, and the angles were measured using Fujifilm OP-A software (Fujifilm Co. Ltd., Tokyo, Japan). Anteroposterior (AP) and lateral whole leg radiographs were obtained with the patients in a standing position, as well as a skyline view of the knee; these radiographs were taken preoperatively and 24 months postoperatively. We took the radiographs 2 years after removal to exclude the influence of the fixation plate. The aFTA was defined as the lateral angle between the femoral and tibial anatomical shaft axes on an AP whole leg radiograph. The weight bearing line (WBL) ratio was defined as the ratio of the distance between the medial edge of the tibial plateau and the intersection point to the length of the tibial plateau. The WBL ratio of the medial tibial edge was considered to be 0% and the lateral tibial edge to be 100%. The medial TPS on the whole leg lateral radiograph was measured as the angle between the posterior slope and a line perpendicular to the tibial shaft axis [10]. We calculated the change in TPS by subtracting preoperative TPS from postoperative TPS. The anatomical lateral distal femoral angle (aLDFA) was defined as the lateral angle between the femoral anatomical axis and the tangent of the femoral condyles, and the anatomical medial proximal tibial angle (aMPTA) was defined as the medial angle between the tibial anatomical axis and the tibial plateau on the coronal view. The joint line convergence angle (JLCA) was defined as the angle between the tangents to the femoral condyles and the tibial plateau. JLCA with varus was represented as a “+” sign.

### Computed tomography

**Fig. 1:**
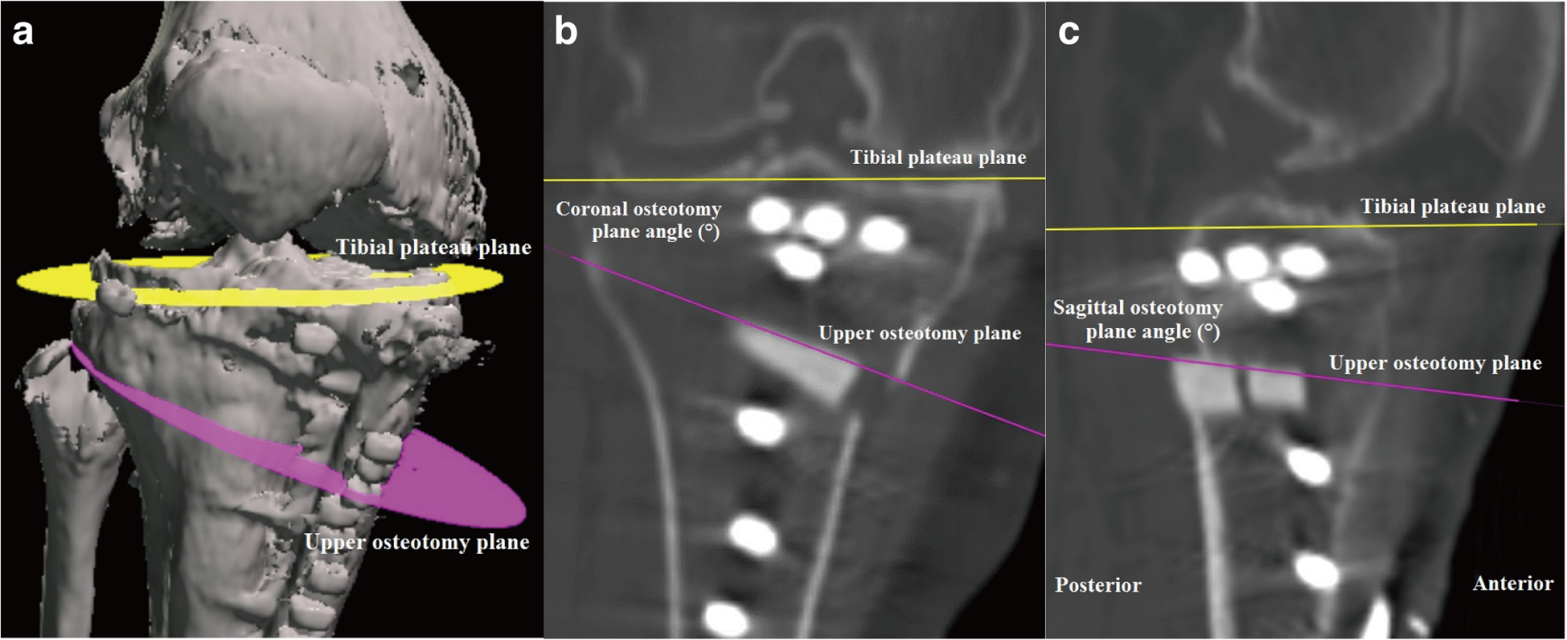
**a** Three-dimensional computed tomography images of the knee joint. **b** The coronal osteotomy plane angle between the tibial plateau and the upper osteotomy planes in the coronal reference plane.** c** The sagittal osteotomy plane angle between the tibial plateau and the upper osteotomy plane in the sagittal reference plane

**Fig. 2:**
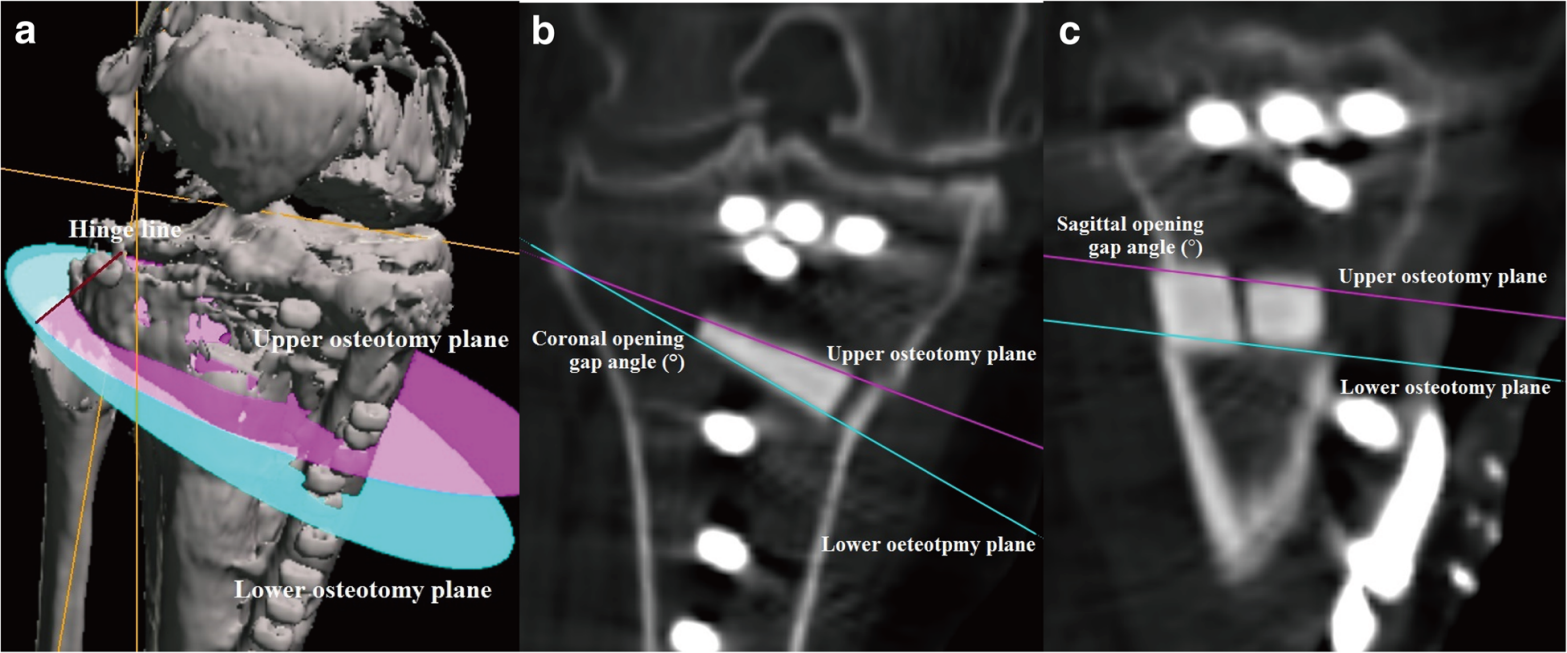
**a** Three-dimensional computed tomography images of the knee joint. **b** The coronal opening gap angle between the upper and lower osteotomy planes in the coronal reference plane. **c** The sagittal opening gap angle between the upper and lower osteotomy planes in the sagittal reference plane

**Fig. 3:**
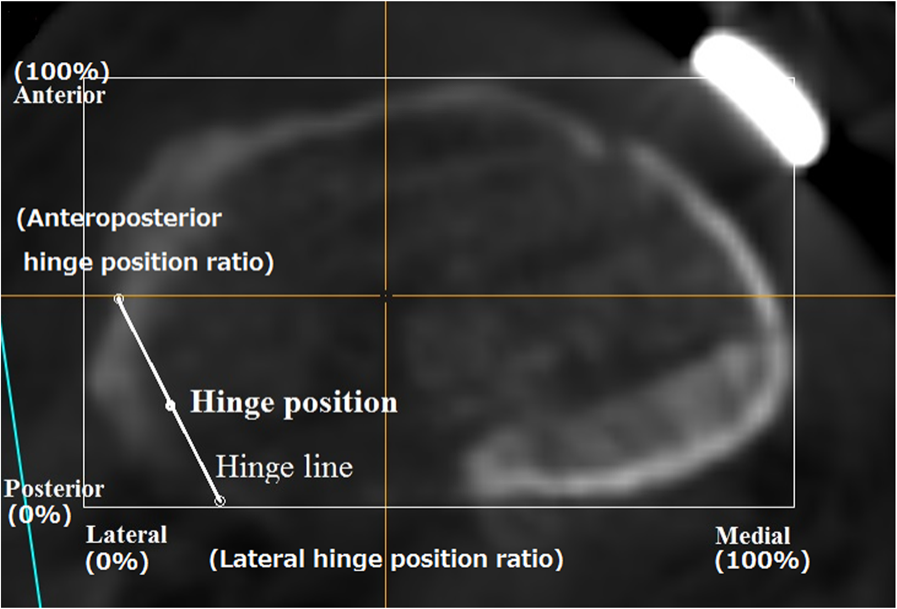
Cross-sectional computed tomography image on the upper osteotomy plane. In the upper osteotomy plane, the midpoint of the line connecting the two points where the hinge line intersects the proximal tibial lateral cortex (hinge position) is displayed on the x-y coordinate system based on the mediolateral and anteroposterior width

Pre- and post-operative CT images of the whole lower extremity were obtained in 1.5-mm-thick slices using a SOMATOM Sensation 16 CT scanner (Siemens, Munich, Germany). The postoperative CT images were taken at 3 months after OWHTO. The analysis was performed using the CT images taken 3 months after OWHTO, because we could not confirm the borders between the osteotomy plane and artificial bones owing to bone union in later images. The data were incorporated into the Orthomap3D navigation software (Stryker, Kalamazoo, MI, USA), which enabled us to select anatomical landmarks and determine 3D linear and angular measurements by simultaneously referring to the sagittal, coronal, and axial planes [10, 11]. To measure each angle, the coronal and sagittal reference planes were set. The sagittal reference plane in the tibia was defined as the plane connecting the medial border of the patellar tendon at the level of the patellar tendon attachment, the middle of the posterior cruciate ligament at the level of the tibial plateau (connected to the Akagi line), and the ankle center [12]. The coronal reference plane in the tibia was defined as the plane perpendicular to the sagittal plane and passing through the middle point of the tibial eminences and the ankle center. The tibial plateau plane was defined as the plane connecting the anterior and posterior limits of the medial tibial plateau and the most lateral point on the line intersecting the lateral tibial plateau and the coronal plane. The upper osteotomy plane was defined as the plane connecting the following three cortical points: the anteromedial corner where the flange intersects with the upper osteotomy line, the posteromedial corner, and the center of the posterior cortex (Fig. 1a). The coronal and sagittal osteotomy plane angles were defined as the angles between the tibial plateau and the osteotomy planes in the coronal and sagittal views, respectively. The angle, which had an anterior-widening proximal tibial fragment, was showed as a positive value (Fig. 1b and c). The lower osteotomy plane, defined as the plane connecting the following three cortical points: the anteromedial corner where the flange intersected with lower osteotomy line, the posteromedial corner, and the center of the posterior osteotomy line (Fig. 2a). The coronal and sagittal opening gap angles were defined as the angles between the upper and lower osteotomy planes in the coronal and sagittal views, respectively. The angle, which is an anterior-opening gap, is showed as a positive value (Fig. 2b and c). When the opened upper and lower osteotomy planes was parallel in the tibial sagittal reference plane, the osteotomy gap in the sagittal view is rectangular. The hinge line was defined as the intersection line between the upper and lower osteotomy planes, as previously described (Fig. 2a) [9]. In the upper osteotomy plane, the midpoint of the line connecting the two points where the hinge line intersects the proximal tibial lateral cortex (hinge position) was displayed on the x-y coordinate system based on the mediolateral and anteroposterior width (Fig. 3). The percentage of the hinge position in the x-axis (lateral hinge position ratio) and y-axis (AP hinge position ratio) were defined. The AP and lateral hinge position ratio of the lateral tibial edge was considered to be 0%, the medial tibial edge to be 100%, the posterior tibial edge to be 0%, and the anterior tibial edge to be 100%. Two of the authors measured the osteotomy plane and opening gap angles in the coronal and sagittal views on CT images.

### Clinical evaluation

Before and 2 years after OWHTO, arc of motion was measured, American Knee Society (AKS) knee and function scores were used to evaluate the degree of knee OA, and the Lysholm knee scoring scale was used to evaluate the conditions of the knee ligament [13, 14]. Clinical evaluations were measured by one of the authors preoperatively and at the 2 year after OWHTO.

### Surgical technique

Preoperative planning anticipated a WBL ratio of 62% on the whole leg radiograph [15]. An arthroscopic examination was performed in all patients before OWHTO. After arthroscopic examination, a thigh tourniquet was applied while the patient was in the supine position. A 4–5-cm incision was made longitudinally at the 4–5-cm medial portion of the anterior ridge of the tibia. The superficial medial collateral ligament (MCL) was released subperiosteally from the medial proximal tibia. The osteotomy plane was obliquely directed from 35 mm distal to the medial tibial plateau using two Kirschner wires parallel to the medial TPS [16, 17]. The osteotomy plane was planned to be parallel to the tibial plateau plane in the sagittal view. The anterior and posterior limits of the medial tibial plateau were superposed under fluoroscopy. Transverse osteotomy was performed from the medial tibia using a bone saw and chisels, while the lateral cortex was left intact of 10 mm as a hinge. The ascending osteotomy was performed under the tibial tuberosity at a 100–120° angle to the horizontal cut in the posterior 2/3 of the tibia in the sagittal view [2]. Under the image intensifier, the osteotomy site was gradually opened by stepwise insertion of 3–5 coupled chisels. The posterior beta-tricalcium phosphate (β-TCP; Olympus Terumo Biomaterials, Tokyo, Japan) was trimmed to a triangular shape the same width as the gap at the posteromedial corner, and the anterior β-TCP was trimmed to 2/3 of the width of the gap in the posteromedial corner. In cases of increased TPS, the posterior soft tissue was released. After the osteotomy and opening, two β-TCP wedges were inserted into the opened gap. The medial gap was filled by two formed β-TCP wedges and fixed with TomoFix and locking screws (DePuySynthes, Solothurn, Switzerland). The superficial MCL was not reattached. A drain was placed after closing the tissue layers.

### Postoperative rehabilitation

All patients received thromboembolism prophylaxis in the form of low molecular weight heparin and venous impulse foot pumps. Patients began active and passive range of motion exercises and straight leg raising exercises on postoperative day 1. Full weightbearing was permitted to begin 1 week after surgery.

## Statistical analysis

Data are expressed as means with 95% confidence intervals. To assess the intraobserver reproducibility and interobserver reliability, the measurements of each angle on postoperative CT were repeated twice by two observers in the first 40 knees. Preoperative and postoperative radiographic and clinical data were compared using t-test and Wilcoxon test. The relationships between the osteotomy plane or osteotomy gap and the TPS were calculated using the Pearson correlation coefficient. The interclass correlation coefficients (ICCs) for the intra- and inter-observer agreements were calculated. IBM SPSS Statistics Desktop for Windows version 21 software (SPSS Inc., Chicago, IL) was used for all statistical analyses. Values of *p* < 0.05 were considered statistically significant. Sample size calculation was performed using G*Power version 3.1.9 (Heinrich-Heine-Universität Düsseldorf, Germany). Priori power analysis (effect size = 0.3, α = 0.05, power = 0.8) resulted in a sample size of 82.

## Results

Patient characteristics are shown in Table 1. Table 2 shows the preoperative and postoperative radiographic and clinical data. The preoperative aFTA of 181.6° was significantly corrected to a postoperative aFTA of 168.5 ° (*p* < 0.001). The preoperative WBL ratio of 14.7% was significantly shifted to the postoperative WBL ratio of 69.8% (*p* < 0.001). The preoperative TPS of 11.9° significantly increased to the postoperative TPS of 12.6° (*p* = 0.002). The arc of motion and AKS knee, AKS function and Lysholm scores before OWHTO were significantly increased than them after OWHTO (*p* = 0.037, *p* < 0.001, *p* < 0.001 and *p* < 0.001, respectively).

**Table 1:** Patient characteristics. Presented as mean (confidence intervals) or number

Variable	Mean (95% CI)
Age (yrs)	65.2 (63.5 to 66.8)
Height (cm)	157.4 (155.5 to 159.3)
Weight (kg)	63.4 (61.0 to 65.9)
Body mass index	25.5 (24.8 to 26.2)
Sex (women / men)	58 / 24
Side (Left / Right)	38 / 44
Ahlbäck grade 0/1/2	1 / 61 / 20

**Table 2.** Preoperative and postoperative radiographic and clinical data. Presented as mean (confidence intervals)

*p*-value	Preoperation	2 years after surgery
aFTA (°) < 0.001^a^	181.6 (180.8 to 182.5)	168.5 (167.8 to 169.2)
WBL ratio (%) < 0.001^b^	14.7 (11.3 to 18.2)	69.8 (66.6 to 73.0)
TPS (°) 0.002^a^	11.9 (11.0 to 12.7)	12.6 (11.7 to 13.5)
aLDFA (°) 0.289^a^	81.3 (80.7 to 81.9)	81.1 (80.6 to 81.6)
JLCA (°) < 0.001^a^	4.6 (4.2 to 5.0)	3.6 (3.2 to 4.0)
aMPTA (°) < 0.001^b^	85.3 (84.3 to 86.2)	95.1 (94.0 to 96.3)
Arc of motion (°) 0.037^b^	125 (122 to 128)	128 (125 to 131)
AKS knee score (point) < 0.001^b^	51 (49 to 53)	86 (85 to 88)
AKS function score (point) < 0.001^b^	72 (69 to 75)	97 (96 to 99)
Lysholm score (point) < 0.001^b^	47 (44 to 50)	89 (87 to 91)

*aFTA* anatomical femorotibial angle, *WBL* weightbearing line, *TPS* tibial posterior slope, *aLDFA* anatomical lateral distal femoral agle, *JLCA* joint line convergence angle, *aMPTA* anatomical medial proximal tibial angle, *AKS* American Knee Society

^a^t-test

^b^Wilcoxon test

Table 3 shows the postoperative radiographic and CT data. The change in TPS was 0.9°. The coronal osteotomy plane angle was 16.6°, and the sagittal osteotomy plane angle was 6.2°. The osteotomy plane angle, which had an anterior-widening proximal tibial fragment in the sagittal view, had a positive value in 78% of knees (64/82). The coronal opening gap angle was 13.7°. The sagittal opening gap angle was − 0.7°. The opening gap angle, which showed the small posterior-opening in the sagittal view, had a negative value in 49% of knees (40/82).

**Table 3:** Postoperative radiographic and computed tomography data. Presented as mean (confidence intervals) or number (%)

Variable	Mean (95% confidence intervals)
Change in TPS (°)	0.9 (0.4 to 1.3)
Coronal osteotomy plane angle (°)	16.6 (15.5 to 17.7)
Sagittal osteotomy plane angle (°)	6.2 (4.4 to 8.1)
Positive / negative value (knees)	64 (78%) / 18 (22%)
Coronal opening gap angle (°)	13.7 (13.0 to 14.5)
Sagittal opening gap angle (°)	−0.7 (−1.4 to 0)
Positive / negative value (Knees)	40 (49%) / 42 (51%)

Sagittal osteotomy plane angle with an anterior-widening proximal tibial fragment is showed as a positive value

Sagittal opening gap angle with an anterior-opening gap is showed as a positive value

*TPS* tibial posterior slope

**Fig. 4:**
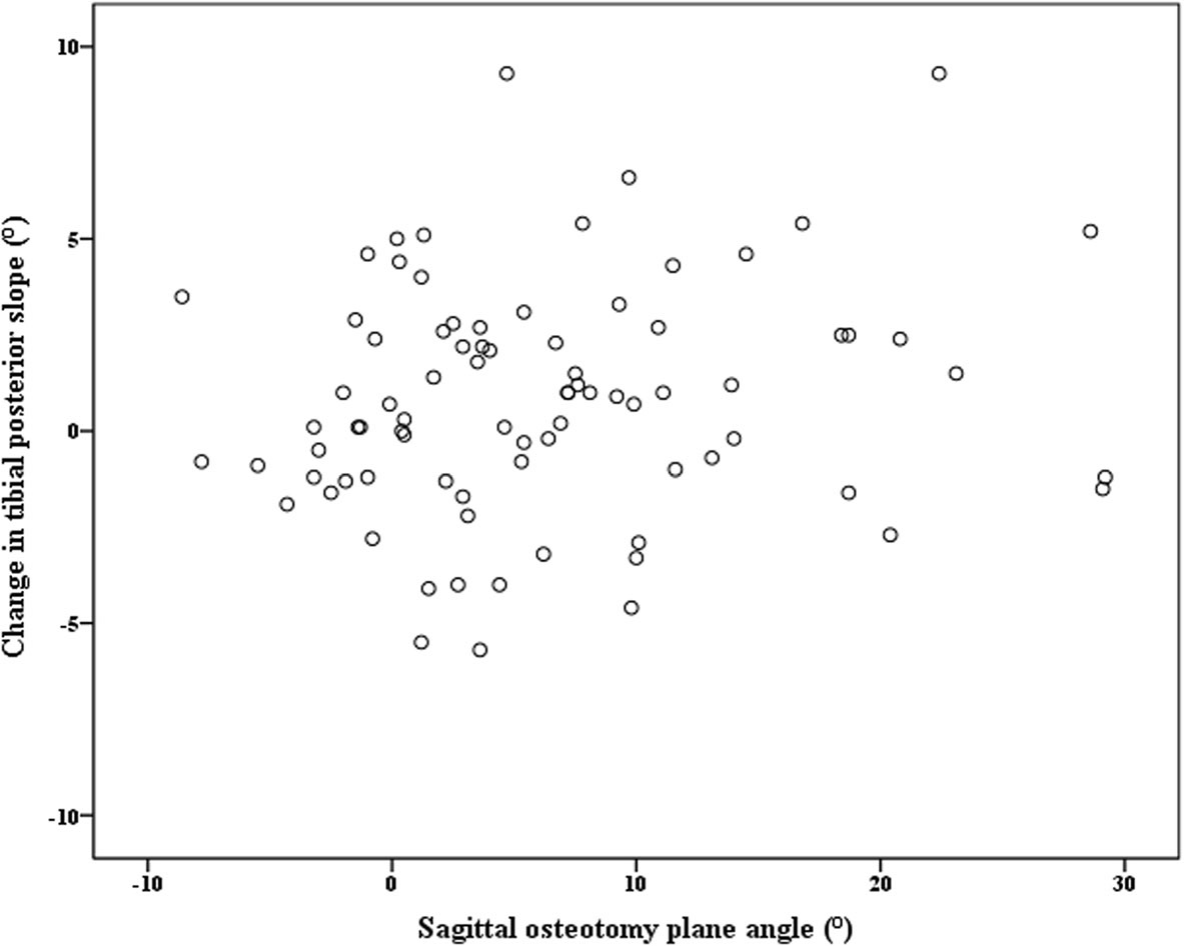
Scatter graph showing the relationship between sagittal osteotomy plane angle and change in tibial posterior slope. The sagittal osteotomy plane was not related with the tibial posterior slope

**Fig. 5:**
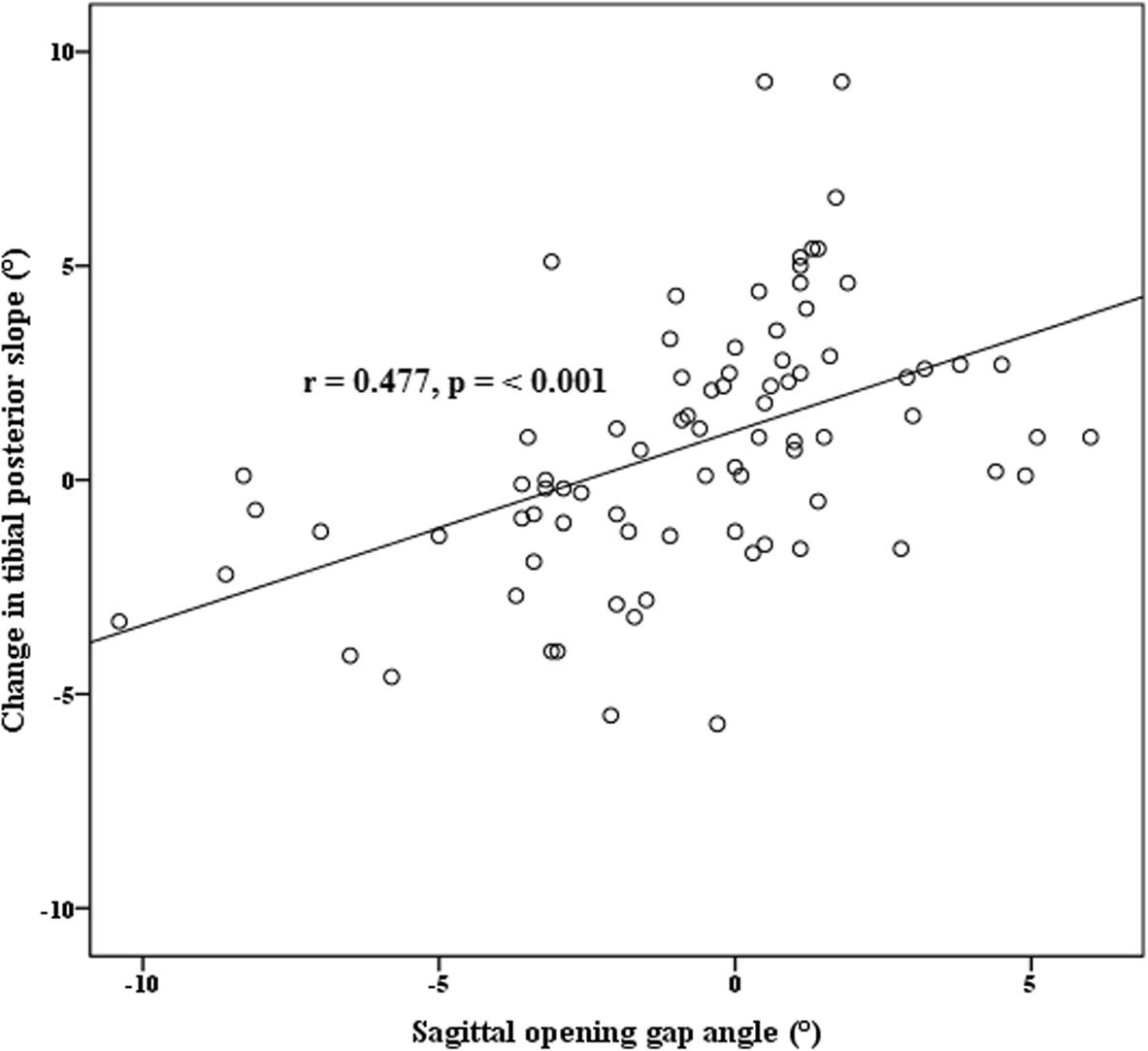
Scatter graph showing the relationship between sagittal opening gap angle and change in tibial posterior slope

There was no significant difference between the sagittal osteotomy plane angle and the change in TPS. The sagittal opening gap angle and the AP hinge position ratio were significantly correlated with the change in the TPS (*r* = 0.477 *p* < 0.001 and *r* = − 0.342, *p* = 0.002, respectively) (Figs. 4 and 5).

**Fig. 6:**
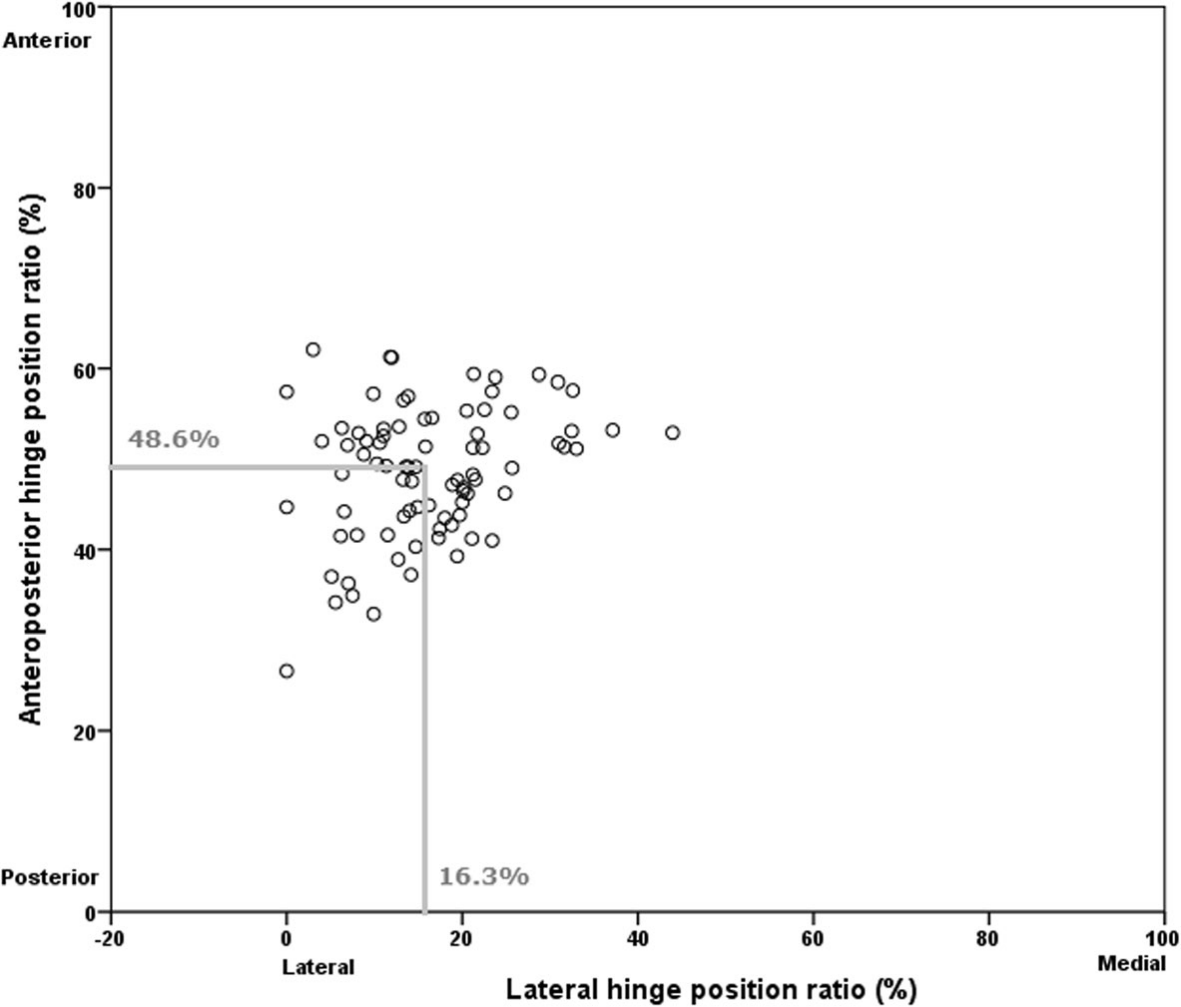
Scatter graph showing the relationship between anteroposterior hinge position ratio and lateral hinge position ratio

The ICCs of the intra-observer agreement for the AP and lateral hinge position ratios were 0.902–0.888 and 0.907–0.838, respectively. The ICCs of the inter-observer agreement for the AP and lateral hinge position ratios were 0.875 and 0.833, respectively. The hinge position was located at a mean of 16.0% (10.0 mm) from the lateral tibial edge, and a mean of 48.6% (21.9 mm) from the posterior tibial edge on the upper osteotomy plane (Fig. 6).

## Discussion

The sagittal osteotomy plane angle with an anterior-widening proximal tibial fragment was not related with increased TPS. The sagittal opening gap angle and the AP hinge position ratio were significantly correlated with the change in the TPS. The hinge position was located a mean of 16% from the lateral edge and a mean of approximately 50% from the posterior edge on the upper osteotomy plane of the proximal tibia.

In the current study, the mean sagittal osteotomy plane angle in 3DCT was 6.2° and 78.0% of knees had an anterior-widening proximal tibial fragment, a sagittal osteotomy plane angle with an anterior-widening fragment was not related to an increased change in TPS. Meanwhile, Lee et al. [4] reported that the mean sagittal osteotomy plane angle in lateral radiograph was 15.1°, and 87.1% of knees had an anterior-widening proximal tibial fragment. They also showed that the sagittal osteotomy plane angle was positively correlated with the change in TPS. The relationship between the sagittal osteotomy plane angle with an anterior-widening fragment and increased change in TPS was different between our and their studies [4]. Possible reasons for the difference between our and their studies might be related to the smaller mean value in the sagittal osteotomy plane angle, the difference in measurement method between radiograph and CT, or the difference in the treatment of the MCL and soft tissue. In addition, the operative technique that the anterior and posterior limits of the medial tibial plateau under fluoroscopy and inserted two K-wires in the osteotomy plane parallel to the medial TPS was superposed might be one of the reasons.

Regarding to the hinge position in the sagittal view, a previous study using 19 knees on 3D CT defined the direction of the hinge line compared with the AP axis on 3D CT axial view as the hinge axis, and reported that the hinge axis was located 4.9° posterolaterally compared with the AP axis [9]. They concluded that a posterolateral hinge position led to a significant increase in TPS. We showed the hinge position ratio using a coordinate system, which was calculated from the hinge line and the lateral tibial cortex on the upper osteotomy plane. This method is applicable to three-dimensional (2D) AP radiographs. Unlike their finding, we found that the hinge position ratio was located a mean of approximately 50% from the anterior edge on the upper osteotomy plane of the proximal tibia. We performed osteotomy from the medial proximal tibial surface after the release of the superficial MCL with an intact pes anserinus [17]. In addition, the release of the posteromedial soft tissue from the periosteum was adjusted according to the tightness of the posteromedial gap. Therefore, an adequate release of the MCL and posterior soft tissues might enable the hinge position to be located centrally in the sagittal view.

The hinge position ratio in the coronal view was located to the medial side of 16% from the lateral cortex on the upper osteotomy plane. A preoperative planning was measured on the AP standing whole leg radiograph of 2D, and the posteromedial corner was opened as a hinge of the lateral cortical edge. A previous study [9] and our results showed that the medial opening of the proximal tibia had been made at the axis of the hinge line where the upper and lower osteotomy planes intersected. Therefore, our findings showed that the postoperative WBL ratio moved more laterally than template during the preoperative planning. In addition, unintended decrease of the JLCA [18, 19] might result in a little more laterally postoperative WBL ratio. The medial movement of hinge position from most lateral had also affected a little more WBL ratio than 62% after OWHTO. Conversely, the hinge position near to the lateral cortical edge would more affect the risk of the lateral cortical fracture.

The opening gap angle and hinge position ratio in the sagittal view was correlated with an increased change in TPS. One study to 12 knees using cadavers [8] investigated whether the variable hinge position actually affected the change in TPS, and concluded that unintentional change in TPS could be avoided if a true lateral hinge position was achieved by performing a complete posterior osteotomy and inspecting the osteotomy using the gap ratio. They also stated that the osteotomy of the proximal tibia using the lateral location as a cortical hinge affected the change in TPS less than using the posterolateral location as a cortical hinge. In our study, the lateral hinge position ratio was located a mean of approximately 50% from the posterior edge. However, our findings were consistent with previous findings that the posterolateral hinge position led to an increase in TPS after OWHTO, and the posterior hinge position was related to anterior-opening gap. The control of AP hinge position must minimize the change in TPS. Conversely, the mean sagittal opening gap angle in our study was − 0.7° and the upper and lower osteotomy were almost opened parallel on the sagittal reference plane in the tibia. The aiming of the horizontal cut in the posterior 2/3 of the tibia and anterior gap of 2/3 of the posterior gap in the sagittal view enabled us to make an almost paralleled opening gap in the sagittal view [2].

We acknowledge the limitations of this study, which the various angles between the two planes were measured on 3D CT with the patients in the supine position, but the alignment was measured on AP whole leg radiographs with the patients in the standing position. In addition, the hinge position, osteotomy plane and opening gap angles were measured using the CT images taken 3 months after OWHTO, but radiographic and clinical evaluations were performed at 2 years after OWHTO.

## Conclusions

Contrary to our expectation, the osteotomy plane did not need to be parallel to the tibial plateau plane in the sagittal view. However, the osteotomy gap should be rectangular in the sagittal view. The hinge position located nearly in the center of the sagittal view.

## Abbreviations

3DCT: Three-dimensional computed tomography
aFTA: Anatomical femorotibial angle
AKS: American Knee Society
aLDFA: Anatomical lateral distal femoral angle
aMPTA: Anatomical medial proximal tibial angle
AP: Anteroposterior
CT: Computed tomography
ICCs: Interclass correlation coefficients
JLCA: Joint line convergence angle
MCL: Medial collateral ligament
OWHTO: Open wedge high tibial osteotomy
TPS: Tibial posterior slope
WBL: Weight bearing line
β-TCP: Beta-Tricalcium phosphate
